# The Role of Host Glycobiology and Gut Microbiota in Rotavirus and Norovirus Infection, an Update

**DOI:** 10.3390/ijms222413473

**Published:** 2021-12-15

**Authors:** Nazaret Peña-Gil, Cristina Santiso-Bellón, Roberto Gozalbo-Rovira, Javier Buesa, Vicente Monedero, Jesús Rodríguez-Díaz

**Affiliations:** 1 Department of Microbiology, School of Medicine, University of Valencia, Avda. Blasco Ibáñez 17, 46010 Valencia, Spain; nazaret.pena@uv.es (N.P.-G.); cristina.santiso@uv.es (C.S.-B.); rovigoro@uv.es (R.G.-R.); javier.buesa@uv.es (J.B.); 2 Department of Biotechnology, Institute of Agrochemistry and Food Technology (IATA-CSIC), 46980 Paterna, Spain; btcmon@iata.csic.es

**Keywords:** rotavirus, norovirus, gut microbiota, HBGAs

## Abstract

Rotavirus (RV) and norovirus (NoV) are the leading causes of acute gastroenteritis (AGE) worldwide. Several studies have demonstrated that histo-blood group antigens (HBGAs) have a role in NoV and RV infections since their presence on the gut epithelial surfaces is essential for the susceptibility to many NoV and RV genotypes. Polymorphisms in genes that code for enzymes required for HBGAs synthesis lead to secretor or non-secretor and Lewis positive or Lewis negative individuals. While secretor individuals appear to be more susceptible to RV infections, regarding NoVs infections, there are too many discrepancies that prevent the ability to draw conclusions. A second factor that influences enteric viral infections is the gut microbiota of the host. In vitro and animal studies have determined that the gut microbiota limits, but in some cases enhances enteric viral infection. The ways that microbiota can enhance NoV or RV infection include virion stabilization and promotion of virus attachment to host cells, whereas experiments with microbiota-depleted and germ-free animals point to immunoregulation as the mechanism by which the microbiota restrict infection. Human trials with live, attenuated RV vaccines and analysis of the microbiota in responder and non-responder individuals also allowed the identification of bacterial taxa linked to vaccine efficacy. As more information is gained on the complex relationships that are established between the host (glycobiology and immune system), the gut microbiota and intestinal viruses, new avenues will open for the development of novel anti-NoV and anti-RV therapies.

## 1. Enteric Viruses and Their Impact on Human Health

Diarrheal disease was one of the top 10 global causes of death in 2016, being the second most common in low-income countries, as reported by the World Health Organization (WHO) [1]. Acute gastroenteritis (AGE) caused by viral infections is the most common type of diarrheal disease. Enteric viruses such as human noroviruses (NoVs) and rotaviruses (RVs) are one of the most important causes of AGE and are known to cause diarrhea, dehydration, or vomiting among other symptoms, leading to the death of patients in the worst cases. These infections have been associated with the consumption of contaminated food or water, person-to-person transmission via direct contact, exposure to aerosols, or the fecal–oral route [2].

RVs caused the death of 528,000 (465,000–591,000) children less than five years old in 2000 worldwide. This number decreased to 215,000 (197,000–233,000) in 2013 thanks to the introduction of vaccines [3] and as of October 2018, 98 countries have included them in their vaccination programs [4]. Currently, there are four different anti-RV vaccines: Rotarix, Rotateq, Rotasiil, and Rotavac [5]. Although they have lower efficacy in low-income countries, a greater reduction in the absolute numbers of AGE and related deaths has been linked to RV vaccination [6].

RV is a member of the *Reoviridae* family and its genome is fragmented into 11 segments of double-stranded RNA. Each segment encodes one protein, except for segment 11 which encodes two of them. Its genome codes for six structural proteins (VPs, from viral proteins) and six non-structural proteins (NSPs). The virion consists of a core layer made of VP2, an intermediate layer made of VP6, and an outer shell made of glycoprotein VP7 and protease-sensitive protein VP4, which extends from the VP7 shell and elicits neutralization antibodies [7]. RVs are classified into ten species or groups (A-J) based on the genetic diversity of protein VP6 [8]. Groups A, B, and C are the most common species that infect animals, including humans, with group A being the most prevalent. This group is further classified into G and P genotypes depending on the variability of the genes encoding the outer capsid proteins VP7 and VP4, respectively [1]. Globally, the most commonly reported strains are G1P[8], G2P[4], G3P[8], G9P[8], G4P[8], and G12P[8], with G1P[8] being the most prevalent [9].

Countries that have introduced RV vaccination have experienced dramatic decreases in RV infections and transmission, so NoV is now the leading cause of viral AGE. According to the CDC (Centre for Disease Control and Prevention; Atlanta, GA, USA), NoV is responsible for one out of every five cases of AGE that leads to diarrhea and vomiting and causes the death of 50,000 children every year in the USA [10]. Contrary to RV, there are no NoV vaccines available, although some candidates are under development [11].

NoVs belong to the family *Caliciviridae* and its single-stranded, positive-sense RNA genome of 7.7 kb contains three open reading frames (ORF). ORF1 encodes a polyprotein which is cleaved into seven non-structural mature proteins (NS1 to NS7), essential for viral replication. ORF2 encodes the major structural capsid protein VP1 and ORF3 encodes the minor capsid structural protein VP2. VP1 protein is subdivided into two domains, the protruding (P) and shell (S) domains. The P-domain is further subdivided into P1 and P2 domains, the second one having a highly variable sequence. Since it is also located on the surface of the capsid, the P2 domain is believed to be critical for host immune and receptor interaction. Meanwhile, the S domain acts as a scaffold for the RNA [12,13].

NoVs are classified into 10 genogroups (GI-GX) according to the VP1 amino acid sequence [14], with GI, GII, and GIV being the ones able to infect humans. Among these three genogroups, GI and GII are responsible for the majority of cases. Genogroups are further divided into genotypes, with GII.4 being the most frequent cause of NoV outbreaks [12].

## 2. Glycobiology Mediates Enteric Virus/Host Interactions

Carbohydrate binding is a common method many viruses and other microorganisms use to attach to their host cells. As for RV and NoV, several studies demonstrate that histo-blood group antigens (HBGAs) act as their receptors [15,16]. These complex carbohydrates are linked to proteins or lipids on the surface of red blood cells and mucosal epithelia of the respiratory, genitourinary, and digestive tracts, or as free oligosaccharides in biological fluids such as saliva [17]. HBGAs are synthesised from precursors by stepwise addition of monosaccharides, catalyzed by a set of glycosyltransferases coded by three major HBGA gene families: secretor, Lewis, and ABO [17]. The secretor gene codes for an α-1,2 fucosyltransferase (FUT2), the Lewis gene codes for an α-1,3 or α-1,4 fucosyltransferase (FUT3), while the ABO family codes for two glycosyltransferases (A and B enzymes) [18].

**Figure 1:**
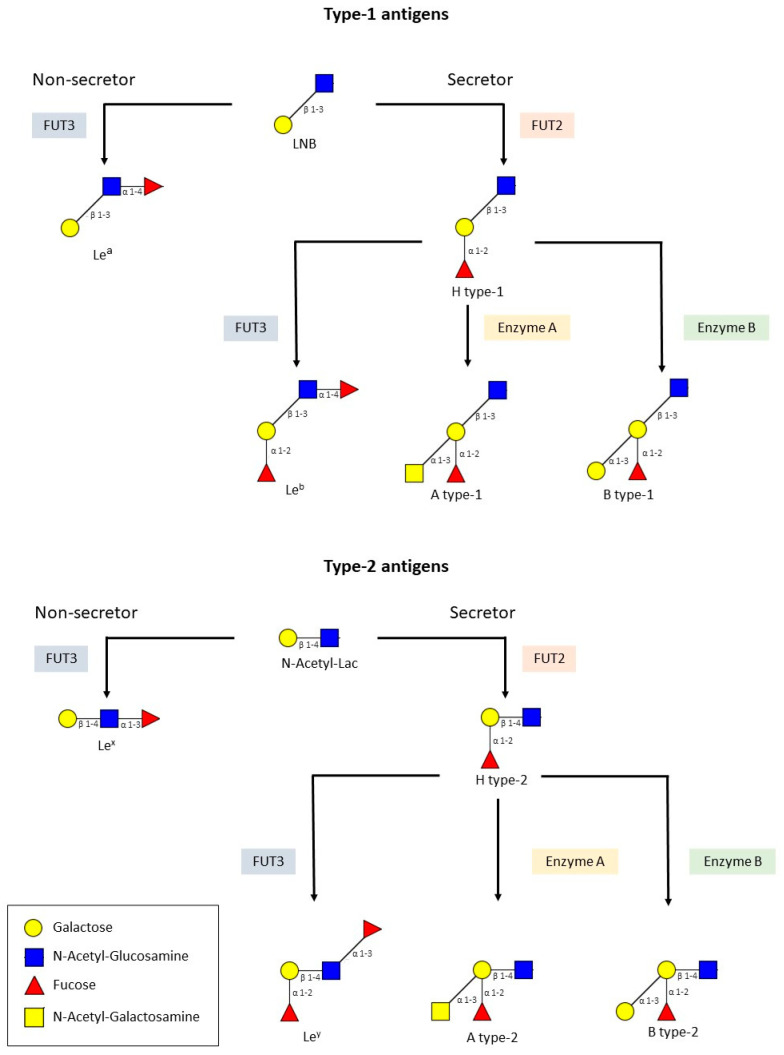
Biosynthesis route for type-1 and -2 HBGAs. The type-1 and the type-2 precursors are further elongated by the fucosyltransferase-2 (FUT2) that adds fucose in α-1→2 to the galactose moiety and the fucosyltransferase-3 (FUT3) that adds fucose in α-1→3/4 to N-acetyl-glucosamine to produce H and Lewis antigens, respectively. Similarly, the A and B enzymes elongate the sugar chains by attaching an N-acetyl-galactosamine or galactose in α-1→3 to the galactose moiety, producing the A and B blood groups, respectively.

The type-1 (galactose-β-1→3-N-acetyl-glucosamine, lacto-N-biose) and the type-2 (galactose-β-1→4-N-acetyl-glucosamine, N-acetyl-lactosamine) precursors act as a substrate of the FUT2 enzyme, which modifies them by the addition of an L-fucose on the galactose moiety through an α-1→2 linkage, generating type-1 and type-2 H antigens, respectively. However, if it is the FUT3 enzyme that modifies the precursors, Le^a^ (type-1 precursor) and Le^x^ (type-2 precursor) antigens are generated. This modification consists of the addition of an L-fucose to N-acetyl-glucosamine with an α-1→4 linkage in the case of the type-1 precursor or an α-1→3 linkage in the case of the type-2 precursor. The FUT3 enzyme can also act on the H type-1 and -2 antigens generating Le^b^ and Le^y^ antigens, respectively. H type-1 and -2 antigens are also substrates of A and B enzymes, giving A and/or B blood groups as a result [17,18] (Figure 1).

Several mutations in the FUT2 or FUT3 genes lead to a total absence or a decreased function of the corresponding fucosyltransferases. The absence of α-1→2- or α-1→3/4-fucosylated antigens in mucous surfaces and secretions determines the non-secretor and the Lewis negative phenotype, respectively [19]. Therefore, these genetic polymorphisms regulate the presence and absence of certain HBGAs, which in turn affects the interaction, susceptibility, or resistance to pathogens that recognize HBGAs as binding molecules [20,21].

### 2.1. HBGAs and RV

Despite the fact that a substantial amount of research has been carried out in the last few years on the RV mechanisms for host cell attachment, the process is still far from being understood. It is known that the VP4 spike protein (determining the viral P genotype) participates in the process and early studies with some P genotypes from animal origin revealed its interaction with sialic acid, whereas other animal and the human RV were sialic acid-independent and interacted with HBGAs [22,23]. VP4 from RV is post-translationally cleaved into VP8* (glycan-binding domain) and VP5* polypeptides, the VP8* portion being the one responsible for cellular attachment and entry, as well as HBGA binding [24]. The P genotypes would thus determine the pattern of genetic susceptibility. VP8* from P[8], P[4], P[6], P[14], P[11], and P[19] genotypes recognize the secretor HBGAs. Regarding P[8] and P[4] genotypes, there were controversial studies since some determined that they bind the Le^b^ and H type-1 [25,26,27,28], while others report no Le^b^ binding for these genotypes [29,30]. P[6] binds the H1 antigen but was reported not to bind Le^b^ [31], whereas P[19] binds mucin core glycans with the GlcNAc-β-1→6-GalNAc motif and the type-1 HBGA precursor [32]. Other studies also documented that P[9], P[14], and P[25] strains interact with the A antigen [24,33], whereas P[11] interacts with single and repeated N-acetyl-lactosamine, the type-2 precursor glycan [34]. The HBGAs interactions with VP8* have been investigated by X-ray crystallography in some cases, identifying sugar binding pockets which are different from the sialic acid binding site identified in animal RV [30]. The binding site for N-acetyl-lactosamine and A-antigen in P[11] and P[14] genotypes, respectively, is situated in a cleft between two twisted β-sheets of the typical galectin fold in VP8* [33,34]. A second pocket was identified in P[4], P[6], and P[19] genotypes for lacto-N-fucopentaose I (Fuc-α-1→2-Gal-β-1→3-GlcNAc-β-1→3-Gal-β-1→4-Glc) [30,35] and in P[8] for lacto-N-biose and H type-1 antigen binding [29], which is formed by one of the β-sheets and a C-terminal α-helix. This pocket is not able to accommodate the Le^b^ antigen, which contains an extra α-1→4-linked L-fucose. A second glycan-binding site for Le^b^ formed by the edges of two β-sheets in the VP8* structure has been identified in P[4] and P[8] genotypes and validated by crystallography and NMR techniques [27]. This provides evidence that classical techniques to identify the interactions between VP8* and HBGAs (e.g., glycan-binding assays in ELISA-like format) do not always give reliable results. Therefore, these two viral genotypes possess two glycan-binding sites which may reflect an adaptation to different host HBGAs polymorphisms.

Several observational studies have investigated the association between the secretor status and susceptibility to RV infection in vivo [36,37,38,39,40]. Although some discrepancies have been found, most reports have shown that positive secretor status was strongly associated with susceptibility to P[8] and P[4] genotypes [36,41,42]. As for serological studies, higher RV-specific immunoglobulin G (IgG) titres in serum and IgA titres in saliva have been reported in secretors compared to non-secretors [43,44,45,46]. Higher anti-RV antibody titres reflect a larger number of previous infections, making it an indirect marker of susceptibility. A study conducted by Sharma et al. showed that human intestinal enteroids isolated from secretor individuals were more susceptible to RV infection as compared with human intestinal enteroids of a non-secretor individual [8]. Moreover, in experiments performed with porcine enteroids, it was shown that infection with a P[8] human RV strain was enhanced by the presence of H and A antigens [47]. It has been determined that the L-fucose moiety of H type-1 glycan in position α-1→2 does not make contact with VP8* and that the unfucosylated precursor (lacto-N-biose) also binds the P[8] genotype at the same binding pocket [29]. The lack of protein interactions of the HBGAs L-fucose moiety was also reported when analysing the binding of P[9] and P[14] genotypes to the A-antigen by NMR and minimal contacts of L-fucose have been reported for P[4] and P[6] during lacto-N-fucopentaose I binding [35]. However, the presence of L-fucose increases two-fold the affinity of P[8] VP8* to the glycan as measured by surface plasmon resonance [29]. Interestingly, this increase in affinity mediated by fucose residues that do not interact with the protein has been also observed in human galectin-3 binding to HBGAs [48]. Whether this increase in the in vitro affinity might provide an explanation as to why secretor positive individuals have a higher susceptibility to RV infections deserves further research. In this regard, it has to be pointed out that although infection with the P[8) genotype RV takes place in FUT2^-/-^ individuals, it occurs at lower levels, as determined by measuring specific antibody titres [45], and that the soluble unfucosylated H type-1 antigen precursor (lacto-N-biose) has inhibitory properties against P[8] RV infection in vitro [29].

The lack of minor interaction of L-fucose with VP8* in particular cases has led some authors to the conclusion that the secretory L-fucose does not play a relevant role in infectivity, at least for some P genotypes, which would agree with the epidemiological data [49]. Thus, a study by MacDonald et al. suggested that there was no association between secretor status and susceptibility to P[6] RV infection since similar proportions amongst secretors (53%) and non-secretors (47%) was observed [50]. Therefore, the controversy about the role of HBGAs on susceptibility still exists and it is intensified by the fact that, as already mentioned, different techniques employed to determine the interaction of VP8* from different P genotypes to HBGAs (i.e., glycan-binding assays, crystallography, or NMR) have usually rendered distinct or contradictory results.

The initial steps of RV attachment to cells and cell entry also include protein-protein interactions, as well as the fusion of membranes mediated by the VP5* portion of VP4. A comprehensive revision of the steps of RV entry and the triggered signalling pathways has been recently published [22].

### 2.2. HBGAs and NoV

The P2 subdomain found in the P-domain of the VP1 protein from NoVs interacts with HBGAs. Several studies have been made in order to elucidate the recognition pattern of NoVs. Some of them are based on the expression of the P-domain in vitro, which results in dimerization (P dimer) and the formation of P particles that retain HBGA-binding function, while others used virus-like particles (VLPs). These studies have utilized ELISA or haemagglutination-based assays using saliva, human milk, red blood cells, or synthetic oligosaccharides as HBGAs sources. The prototype Norwalk virus (GI.1) recognizes the type A and H secretors, but does not interact with type B secretors and non-secretors; Va387 (GII.4) binds to A, B, and O secretors; MOH (GII.5) and Hiro (GII.12) bind to A and B secretors; and Va207 (GII.9) recognizes Lewis positive secretors and non-secretors (Le^x^ and Le^y^) [18,51]. As for the GII.4 strains Den Haag_2006b and Sydney_2012, Carmona et al. demonstrated that they did not recognize any HBGAs [52]. By contrast, these strains may recognize heparan sulphate or citrate since they are all capable of binding human NoV and may potentially play a role in NoV pathogenesis as cellular receptors/co-factors [53]. GI.3 NoV VLPs show strong binding to blood type A salivary HBGAs, slightly lower binding to blood type O salivary HBGAs, and weakly binding or none to blood type B and AB salivary HBGAs [54].

However, whether the secretor status mediates resistance to NoV infection is yet to be solved. As early as 1977, Parrino et al. observed that some individuals were repeatedly susceptible to Norwalk virus (GI.1) infection, whereas a second group was repeatedly resistant. They postulated that a genetic factor might be responsible for susceptibility to infection [55]. While most studies have shown that non-secretors are protected against GII.4 infection and disease, exceptions have been found since there is some evidence of both asymptomatic and symptomatic infections among non-secretors [56,57,58,59]. The reasons for this are unknown, but they could be related to several causes, including microbiota diversity, which would also comprise HBGA-expressing bacteria, differences between GII.4 variants, general health status, weak-secretor phenotype, or other unidentified host factors. Interestingly, Lin et al. described that secretor patients have prolonged diarrhea, more frequent vomiting, more severe disease, and greater infection transmissibility than non-secretors [60].

## 3. The Role of Bacteria in RVs and NoVs Infection: Studies with Cultured Cells and Animal Models

A large and diverse population of commensal microbes consisting of bacteria, viruses, fungi, and parasites inhabit the gastrointestinal tract. NoV and RV, being enteric pathogens, interact with them, resulting in outcomes either beneficial or detrimental to the host [61,62,63]. The coevolution of the commensal microbiota and their host has resulted in a mutually beneficial condition in which the host can benefit from physiological, metabolic, and immunological regulations provided by the microbiota, while the commensal microbiota depends absolutely on the host for nutrient acquisition and propagation sites. In the regulation of viral infection, commensal microbiota can promote inhibitory effects or viral infectivity through diverse mechanisms [64,65].

### 3.1. Bacteria against Enteric Viral Infections

Several studies demonstrate the beneficial effect of probiotic bacteria against enteric virus infections and many other diseases [66,67,68,69]. Probiotics protect the host from viral infection by modulating gut microbiota composition, enhancing intestinal barrier function, and promoting mucosal immunity [70]. Additionally, they interfere with the binding of the virus to their target cells by competitive exclusion by blocking viral receptors and binding viruses on the surface to promote their elimination in faeces [71].

The presence of *Bifidobacterium adolescentis* inhibits the attachment of human NoV (hNoV) GI.1 VLPs to epithelial cells in vitro [72]. Similarly, *Lacticaseibacillus casei* and *Escherichia coli* Nissle 1917 impaired the attachment of GI.1 P-particles to HT-29 cells [73]. In another study, gnotobiotic pigs colonized with *Lacticaseibacillus rhamnosus* GG and *Escherichia coli* Nissle 1917 were infected with human norovirus from the GII.3 and GII.4 genotypes, and a virus faecal shedding below the limit of detection was observed, indicating significant inhibition on hNoV infection by the colonization of such bacteria [74].

As for RV, *Escherichia coli* Nissle 1917 seemed to reduce diarrhea in gnotobiotic pigs by modulating an immune response [75,76,77,78]. Bacterial flagellin is efficient against RV infection since it induces the production of IL-22 and IL-18 [79]. It has also been proved that *Ruminococcus gauvreauii*, a bacterium that has been isolated from human bile and is therefore likely present at the site of RV infection (the small intestine) can bind RV [80]. This binding might be mediated by HGBA-like substances that are present on the bacterial surfaces. Further experiments employing Caco-2 cells demonstrated that *R. gauvreauii* interferes with RV infection in vitro since a threefold decrease in viral infectivity was found in its presence, demonstrating the anti-RV effect of this bacterium [80].

### 3.2. Microbiota and Promotion of Enteric Viral Infections

Despite the significant evidence available about the role of intestinal-derived bacteria in the inhibition of viral infections, several investigations argued for a role of microbiota in promoting virus infection [81]. This was first demonstrated by Kuss et al. [82] and Kane et al. [83] when using poliovirus, reovirus, and mouse mammary tumour virus (MMTV) for infecting germ-free or antibiotic-treated mice. In these cases, it was observed that a substantial attenuation of infection occurred when compared to infection of microbially-colonized mice. Reconstitution of intestinal microorganisms into antibiotic-treated mice was enough to restore poliovirus pathogenesis [82]. Moreover, intestinal titres of reovirus were substantially reduced in antibiotic-treated, compared with control mice [83]. Similar findings were reported with RV and NoV when antibiotic-treated or germ-free mice were used [84,85], suggesting that microbiota enhances the pathogenesis of multiple families of enteric viruses.

The intestinal microbiota can directly facilitate enteric virus replication by several mechanisms, including stabilization of virions and promotion of virus attachment to host cells. Indirectly, it enhances the infection of enteric viruses by altering the antiviral immune response [86]. Several enteric viruses that benefit from the microbiota bind bacterial surface polysaccharides, resulting in enhanced viral infectivity and pathogenesis. When poliovirus and other members of the *Picornaviridae* family bind to lipopolysaccharide (LPS), a component of the Gram-negative bacterial wall, an increase in thermostability and resistance to inactivation at elevated temperatures and in the presence of dilute chlorine bleach can be observed [87]. LPS can also promote poliovirus attachment to the surface of target cells by facilitating viral binding to its receptor [88]. MMTV can bind LPS as well, which stimulates TLR4, initiating a signalling pathway that results in the production of the immunosuppressive cytokine IL-10, generating a tolerogenic environment that allows viral persistence [89].

There is also evidence that suggests that RV and NoV infection is facilitated by microbiota. Interactions of NoV with members of the intestinal microbiota have been demonstrated, including *Enterobacter cloacae*, *Escherichia coli*, and *Helicobacter pylori* [85,90,91]. These interactions are mediated via HBGA-like carbohydrates expressed on the surface of these bacteria [92,93], although NoV has been reported to bind additional carbohydrate residues widely expressed on microbiota [53]. In vitro, human NoV is able to infect B cells in the presence of HBGA-coated bacteria, and a reduction of viral replication was observed in this model if bacteria were not present. Infection of B cells is restored if cells are incubated with *Enterobacter cloacae*, suggesting that the binding of viral particles to HBGA-coated bacteria enables uptake of the virus into the host cells [85]. It was also observed that HBGA-expressing bacteria, such as some *E. coli* strains, protect NoV VLPs during heat treatment, such as the one accomplished during food processing, facilitating their transmission [93]. Experiments performed in gnotobiotic pigs with transplanted human intestinal microbiota showed that replication of the human NoV GII.4/2006b strain was stimulated by the microbiota, which increased shedding titres and duration [94]; experiments in antibiotic-treated mice demonstrated that microbiota eradication prevents the persistent infection of the murine NoV CR6 strain [95]. It was suggested that the intestinal microbiota limits the IFNλ-dependent innate immunity, allowing NoV persistence. The effects of antibiotics were restricted to the intestine, because when CR6 was administered intraperitoneally or in mice lacking IFNα and IFNβ receptors, viral levels in mesenteric lymph nodes and spleen did not change with respect to control mice [95].

### 3.3. Microbiota and Restriction of Enteric Viral Infections

Recently, it has been shown that microbiota ablation with antibiotics in mice allows for infection with the human RV strain Wa (G1P[8]), which replicates very inefficiently in animals with normal microbiota [96]. These results are in conflict with earlier experiments which demonstrated that microbiota eradication by antibiotics results in reduced infection of murine RV (EC strain), as shown by lower viral shedding in adult mice and diminished diarrhea incidence in mice pups [84]. Nevertheless, viral clearance lasted longer in this model. Furthermore, recent experiments with the murine EDIM strain confirmed that antibiotic treatment and the consequent decrease in intestinal bacterial loads or the use of germ-free mice results in increased RV infection [97], which argues against a positive effect of the microbiota in RV infection. In the experiments with the Wa strain, animals with ablated microbiota and subsequent subjection to self-transplantation of intestinal microbiota partially recovered the resistance to infection, which allowed the identification of bacterial taxa that likely participate indirectly or directly in the restriction of Wa infection in mice [96]. Thus, bacteria belonging to lactobacilli, *Mucispirillum*, *Oscillospira*, and *Bilophila* genera were negatively linked to RV infection in mice. Faecal material transplantation with infants as donors did not restrict infectivity in this model, suggesting that the microbiota from the donors was not able to control RV infection in this model and that mice autochthonous bacteria were needed for the process [96]. Although differences depending on the host and viral strains cannot be excluded, all these data point to the microbiota as a major factor limiting RV replication. In this sense, other studies have also determined the role of specific bacterial taxa from the intestinal microbiota in viral replication. Thus, Shi et al. discovered that the elevated RV resistance of certain colonies of Rag1-KO mice (lacking B and T lymphocytes), which usually tend to develop chronic RV infection, to the murine EC strain was due to elevated levels of colonization by *Candidatus* Arthromitus. This bacterium is a member of the segmented filamentous bacteria (SFB), which are typical in mice but can reach elevated numbers in the immunocompromised Rag1-KO strain [98]. SFB participated in the exclusion of RV from mice by processes that involve direct RV/bacteria contact and other mechanisms that are probably based on an increased enterocyte turnover triggered by SFB.

Data about the negative effects of the microbiota on NoV infectivity are scarcer. Epidemiological studies suggest that supplements of vitamin A had an anti-NoV effect. Studies with murine NoV showed an increased population of intestinal *Lactobacillus* sp. in mice after vitamin A supplementation, and it was postulated that the antiviral effects of these bacteria, which were demonstrated in vitro on RAW264.7 cells, account for reduced NoV infection [99]. However, as mentioned in the last section, the use of other animal models and NoV points to a positive effect of the microbiota in the replication of this viral group. However, it has to be noted that more recent and detailed analysis with antibiotic-treated mice shows that, while the microbiota enhances murine NoV strain MNV-1 infection in distal regions of the intestine, it restricts infectivity in the proximal small intestine [100]. This regionalisation of the effects is mediated by a distinct abundance of bile acid receptors depending on the intestinal location, which are involved in triggering an anti-NoV IFNλ response that was enhanced by bacteria in the proximal intestine. Microbial modification of bile acids in the small intestine thus has an effect on murine NoV infection at different intestine locations. Inoculation of antibiotic-treated animals with *Clostridium scindens*, a bacterium known to transform primary bile acids into secondary bile acids (that were lowered in antibiotic-treated animals), restored viral inhibition in the proximal small intestine, although it did not enhance infection in the distal gut [100].

It appears that specific members of the microbiota possess restrictive traits to enteric viral infection, at least for RV, whereas evidence for NoV is weaker. Immune regulation is emerging as the mechanism underlying this phenomenon and it is extended to viruses that do not target the gut. Specific bacterial taxa have also been described in the prevention of viral diseases such as encephalomyocarditis virus (EMCV) systemic infection in mice, in which antibiotic treatment also exacerbated infection [101]. Contrarily to other intestinal bacteria tested, *Blautia coccoides* (a former member of the *Ruminococcus* genus) was identified among members of the gut microbiota as a bacterium able to restrict systemic EMCV replication in monocolonized mice. In animals carrying *B. coccoides*, the capacity of macrophages for inducing IFNβ, which protects against EMCV, was restored [101]. Type I IFN (IFNβ) plays a pivotal role in the response against viral pathogens. Lack of bacteria leads to a weaker innate immune response which is characterized by low expression of IFNβ, which hampers an effective macrophage antiviral response [102]. Several bacterial components or derived metabolites are involved in priming host innate immunity against respiratory viruses through IFNβ production, such as the lipo-oligosaccharides from *Bacteroides* in influenza infection [103] or acetate (and propionate or butyrate) from gut microbial metabolism, which is per se able to induce an IFNβ response in the lungs of mice and protect them from the respiratory syncytial virus when supplemented in drinking water [103].

The above-presented studies confirm the influence that the microbiota has over enteric viral infections, as well as over other non-enteric viruses. However, the mechanisms by which this takes place are not well-understood yet for RV and NoV, and new studies are needed in order to gain knowledge about them. Similar to NoV, few examples about the disclosure of in vivo mechanisms of microbiota restriction of infectivity are known in RV. TLR4, recognizing bacterial flagellins, and the NLR-C4 component of the inflammasome are crucial for the production of IL-22 and IL-18, which are important in protection against RV [79]. In the studies conducted by Schnepf et al., a pivotal role for IL-22 induced by the microbiota in its limiting effects against RV infection was also established [97]. It was shown that microbiota depletion resulted in reduced IL-22 production, and that protection against RV can be achieved by IL-22 administration. In this case, IL-22-mediated protection did not involve IFN, because it was also found in mice lacking the transcriptional factor STAT1, which increases the expression of interferon-stimulated genes [97]. Mice treated with antibiotics in which the human RV Wa strain was able to replicate also presented alterations in the expression of genes related to the immune and inflammatory response, such as IL-1β and CXCL15, and the FUT2 enzyme [96], but the relevance of these facts in RV infection has to be evaluated.

Another plausible mechanism that the microbiota could use to restrict infection implies secretory immunoglobulin A (sIgA). The sIgA molecules are secreted into the intestinal lumen, where they attach antigens and act as the first barrier of mucosal defence. The gut microbiota has been shown to be able to regulate IgA production, and the level of IgA in the gut is considerably decreased in germ-free mice [104]. Other ways by which microbiota members could mediate RV protection include direct attachment of RV particles to bacteria, as hypothesized for SFB [46], or have been described for some probiotics. These interactions may be promoted by HBGA-like molecules that can be presented on bacterial surfaces [92]. The consequences derived from this interaction may differ between diverse viral groups and while some enteric viruses, such as NoV or poliovirus, may benefit from them via enhancement of virion stability or target cell attachment [82,85,93], they can also mediate virus sequestration on the bacterial surface and/or competition with the viral binding molecules present at the surface of host cells.

## 4. Microbiota and Enteric Viruses, Studies in Humans

Very few studies have addressed the relationships between gut microbiota and the infection of enteric viruses in humans. Most of the results linking different bacterial taxa to viral infection are derived from vaccination trials, in which the intestinal microbiota analyses have been linked to vaccine outcomes (i.e., RV vaccines (RVVs)). Microbiota composition varies depending on the population [105] since it is affected by many factors including nutrition [106], sex, age, genetics, and health status [107], and these vary greatly between low-income and high-income countries. Such differences could be some of the reasons why RVVs have significantly lower efficacy in low-income countries [108,109].

However, another important reason that could explain such differences is related to the prevalence of Lewis negative individuals in Asia, Latin America, and African countries [58,110], where P[6] is the prevalent genogroup [41] since it recognizes Lewis negative antigens [31]. Moreover, RVVs do not include the P[6] genotype, so this could explain the lower RVVs efficacy in those areas.

RVVs have dramatically reduced the morbidity and mortality of AGE caused by RV infection [3]. Understanding the mechanisms implicated in the reduced efficacy of RRVs is relevant since even small improvements in vaccine efficacy might increase the number of children’s lives saved by hundreds of thousands during the coming years [111]. Although the reasons for variations in efficacy are not fully understood, they are thought to be differences in co-infections with other enteropathogens at the time of vaccination, gut microbiota composition, and HBGAs genotype [107]. Studies in Africa and Asia using Rotarix and Rotateq vaccines have been conducted in order to elucidate gut microbiota differences (by means of 16S rDNA sequencing) between RVVs responders and non-responders. Rotarix consists of a human attenuated single strain (G1P[8]) and Rotateq includes five bovine-human reassortant strains (G1, G2, G3, G4, and P[8]) [6].

Harris et al., using the Rotarix vaccine, demonstrated that intestinal microbiota differs significantly between RVVs responders and non-responders in Ghana [112]. Responders were considered the ones that had anti-RV IgA antibodies ≥20 IU/mL after vaccination. The study found that children that responded to RVVs had abundant counts of bacteria from the *Bacilli* phylum, especially *Streptococcus bovis*, while the non-responders presented abundant numbers of *Bacteroidetes* phylum, specifically *Bacteroides* and *Prevotella* species. Moreover, the study showed that the *Enterobacteria*/*Bacteroidetes* ratio was significantly higher in vaccine responders as compared to non-responders. In addition, responders had more microbiota similarities with Dutch children (assumed to be RVVs responders, in line with clinical trial data demonstrating a >90% RVVs seroconversion rate in northern European countries) than with non-responders [112]. This group conducted similar research with Pakistani infants, also concluding that microbiota varies significantly between RVVs responders and non-responders. They determined that the relative abundance of Gram-negative bacteria such as *Serratia spp.* and *Escherichia coli* correlated positively with RVVs response as compared to non-responders [113]. Researches hypothesised that differences in RVVs efficacy are due to *Bacteroides*, present in more abundance in non-responders since they have LPS that differs from that present in *Enterobacteriacae*. LPS from *Bacteroides* species have been demonstrated to inhibit the stimulation of inflammatory cytokines in vitro using the LPS from *Enterobacteriacae* as a reference [111]. In a similar way, a relative abundance of flagellin-producing bacteria may enhance the innate and subsequent adaptive immune responses to RVVs [79]. In opposition to this, in a study carried with children that had received Rotarix in Zimbabwe and where a very low percentage of vaccine take was observed, faecal microbiota analyses showed that *Bacteroides thetaiotaomicron* was the only bacterium that correlated with high specific IgA titres (responders) [114]. Another hypothesis, given that vaccines contain a live attenuated virus, is that bacteria present in responders might be expressing HBGAs or glycans needed for RV replication [24]. However, other studies conducted with Indian infants showed no significant differences in microbiota composition between responders and non-responders [115]. The researchers hypothesized that the discrepancies in both studies regarding differences in microbiota composition between responders and non-responders could be due to differences in methodology (next-generation sequencing versus microarray) or baseline microbiota composition. As for the Rotateq vaccine, a study carried out with Nicaraguan children determined no statistically significant differences in the microbiome composition between RVVs responders and non-responders [28]. However, the sample size of these studies is small, so further research is advisable in order to have more reliable results.

A recent study evaluated whether microbiota modification by the use of broad- and narrow-spectrum antibiotics had an effect on immunization with Rotarix in adults [116]. Although the experimental groups did not differ in terms of total IgA produced, the narrow spectrum group showed a boost in IgA at day seven post-vaccination (basal levels of anti-RV IgA were high in the vaccination group) and the viral shedding was increased in both groups treated with antibiotics. Differences in the microbiota composition in faeces were evident between the groups and correlations between enrichment in *Bacteroides* populations at the boost at day seven were observed and several taxa (*Prevotellaceae*, *Cloacibacillus everynsis*, and Proteobacteria members such as *Escherichia* and *Shigella*) were associated with increased viral shedding. Antibiotic treatment had no effect on the immunogenicity of other systemic vaccines applied (pneumococcal and tetanus vaccine) [116]. These results highlight the fact that targeting the microbiota could be an alternative strategy to enhance RVVs efficacy, although the effectiveness in children still needs further investigation.

None of the above discussed studies considered the secretor status in the vaccine efficacy. Other studies determined that anti-RV IgA seroconversion rates after Rotarix vaccination differed significantly depending on salivary HBGA phenotype, having the lowest rate of seroconversion (non-responders) infants who were non-secretors [117,118,119]. This finding is consistent with in vitro data, which demonstrated that P[8] strains interacted with H type 1 antigens [29] (and Le^b^ depending on the author [26,27]; these two are carbohydrates expressed only in individuals with functional FUT2 alleles). Thus, differences in HBGA expression may be responsible for some of the discrepancies in the level of protection detected for RVVs in low-income and high-income countries. Other studies have applied 16S rDNA sequencing to analyse the intestinal microbiota of groups of volunteers, examining the susceptibility to RV and NoV measured as the level of salivary NoV and RV-specific IgA, and performing FUT2 genotyping [45]. The results showed that all three factors (gut microbiota, FUT2 genotype, and susceptibility to RV and NoV) are interconnected. It was also found that certain bacterial genera, such as *Ruminococcus*, correlated negatively with the susceptibility to RV and NoV, while *Akkermansia*, an intestinal mucin degrader, correlated positively with RV IgA titres [45]. In mice pups infected with RV, a shift in the ileal microbial populations was observed, with increased levels of mucin degraders such as *Akkermansia* and *Bacteroides* [120]. It was postulated that the observed release of mucin during infection may favour this species, whose glycan-degrading activities on mucin create in turn a glycan environment more favourable for RV infection [120].

An ex vivo study analysed the bacterial groups that were interacting with RV in stool samples from children suffering RV (G1P[8]) diarrhea by flow cytometry followed by 16S rDNA sequencing [80]. This study also allowed the identification of *Ruminococcus* as RV-interacting bacteria. As already mentioned, a species of this group (*R. gauvreauii*) was shown to inhibit RV infection in vitro [80]. This, together with the correlation *Ruminococcus*-anti-RV IgA in humans and the fact that higher *Ruminococcus* numbers are found in healthy children compared to children with RV diarrhea [121], postulates these bacteria as likely players in the cross-talk bacteria-virus-host. Similar studies conducted with individuals suffering AGE caused by NoV will certainly aid in identifying bacterial taxons that interact with these viruses in stools. However, whether this interaction has some relevance in the infection process needs further investigation.

All these findings may help to improve RVVs performance in such a way that they have higher efficacy in low-income countries, preventing tens of thousands of RV-related deaths per year. However, differences in the conclusions drawn from the microbiota analyses are evident, and standardized and controlled methods (e.g., sampling and DNA extraction techniques, bacterial 16S rDNA sequencing platforms, microbial composition analysis methods, etc.) are needed to get a clearer picture.

Regarding vaccines, a different situation is found for NoV. Since up to now there is no NoV vaccine available, differences in vaccine efficacy depending on differences in microbiota composition cannot be studied. Although no NoV vaccine is commercially available, a few of them are in clinical trials [11]. The candidate furthest along in the development pipeline is developed by Takeda Pharmaceuticals. It is a bivalent (genotypes GI.1/GII.4), intramuscular VLP vaccine, currently in phase IIb. The vaccine failed to significantly prevent acute gastroenteritis. However, it reduced severe diarrhea and vomiting [122]. This bivalent vaccine was well tolerated and immunogenic, and the antibodies generated elicited HBGA-binding blocking activity [122]. As for secretor status, secretor and non-secretor individuals responded similarly to the first dose of vaccine [123]. Such a genetic difference in the small intestine is unlikely to have a large impact on vaccine immunogenicity, since most VLP-based vaccines are designed for parenteral administration, thus avoiding the mucosa.

Some clinical trials have studied the relationship between probiotic bacteria and their influence on enteric virus infections. While in vitro assays and studies in animal models have helped to determine probiotic strains with antiviral activity that can be useful in the treatment of RV infections, there is a large controversy in terms of its beneficial effects in humans. Few clinical trials have studied the influence of probiotics in RV infections and many differences can be found. Some of them determined that probiotic treatment for patients with RV-related diarrhea produces shorter diarrhea duration, less RV shedding, faster improvement in stool consistency, and fewer defecation times [124,125,126,127,128,129,130,131,132,133], while only two of them found vomiting reduction [130,134]. One of them even concludes that probiotics reduce the risk of nosocomial RV gastroenteritis [135]. Contrarily, almost half of the analysed clinical trials determined that the intake of probiotics does not produce any improvement in RV-related diarrhea symptoms [136,137,138,139,140,141,142,143,144] (Table 1). The number of subjects enrolled in these clinical trials, the probiotic used, application methods, doses, and the way in which the effects are measured are possible factors affecting the results, for which, again, more standardized and controlled trial conditions are required to assess the efficacy of probiotics in viral AGE.

## 5. Conclusions and Perspectives

Many enteric viruses, such as NoV and RV, have developed mechanisms to continue infecting the host in the presence of a healthy gut microbiota, even to take advantage of it in some cases. However, more studies are desirable in order to have a better understanding of differences in gut microbiota composition that affect RVVs efficacy, and how these differences impact possible anti- and pro-viral mechanisms. Therefore, identification of key bacteria that correlate with RVVs efficacy could be important for designing future vaccines in countries where RVVs have less effectivity [147]. Such bacteria could be also used as biomarkers for vaccine efficacy and interventions that modify the microbiota composition in order to increase it could be envisaged [147].

As for NoV, there is still controversy regarding the role of secretor status in NoV infection. New experiments based on human enteroid models that mimic the human intestinal epithelium could be performed. Therefore, libraries of enteroids generated from individuals with different FUT2, FUT3, and ABO polymorphisms may provide important information on how secretor, Lewis status, and other HBGAs affect NoV infection. There is also a great need for the development of the NoV vaccine. The one being developed by Takeda Pharmaceuticals is currently in phase IIb, and it is based on VLPs. If attenuated NoV vaccines are developed, testing whether their efficacy varies depending on host glycobiology and microbiota will be necessary.

It can be concluded that this is a fast-evolving research field where the complex interactions between the enteric pathogens RV and NoV with the host glycobiology and the gut microbiota are starting to be elucidated. The knowledge earned about these interactions will allow the scientific community to improve the prevention strategies against these viruses as well as to design novel therapeutic approaches for the management of infected patients.

## Figures and Tables

**Table 1:** Effect of probiotics in the treatment of RV in clinical trials.

Microorganism(s) ^a^	Effect	References
*Lactobacillus plantarum*	Diminished diarrhea duration Diminished number of defecation times Fewer patients with diarrhea	[124]
*Bifidobacterium longum* BORI *Lactobacillus acidophilus* AD031	Diminished diarrhea duration	[125]
*Lactobacillus rhamnosus* GG	Diminished diarrhea duration Faster improvement in stool consistency Diminished number of defecation times	[145]
*Saccharomyces boulardii* (yeast)	Diminished diarrhea duration Shorter hospitalization	[126]
*Lactobacillus acidophilus* *Lactobacillus paracasei,* *Lactobacillus bulgaricus* *Lactobacillus plantarum* *Bifidobacterium breve* *Bifidobacterium infantis* *Bifidobacterium longum* *Streptococcus thermophilus.*	Diminished diarrhea duration Diminished number of defecation times Faster improvement in stool consistency	[127]
*Enterococcus faecalis* *Clostridium butyricum* *Bacillus mesentericus* *Lactobacillus sporogenes*	Diminished number of defecation times Diminished diarrhea duration Diminished duration of rotaviral shedding	[128]
*Bifidobacterium lactis* Bb12 *Streptococcus thermophilus* TH4	Diminished RV shedding	[129]
*Lactobacillus rhamnosus*	Diminished diarrhea duration	[130]
*Lactobacillus casei* *Lactobacillus acidophillus* *Saccharomyces boulardii*	Diminished diarrhea duration Diminished number of defecation times Diminished vomiting	[131]
*Lactobacillus rhamnosus* 19070–2 *Lactobacillus reuteri* DSM 12246	Diminished RV shedding	[132]
*Lactobacillus rhamnosus* GG	Diminished RV shedding Diminished diarrhea duration	[133]
*Lactobacillus acidophillus* *Lactobacillus rhamnosus* *Bifidobacterium longum* *Saccharomyces boulardii*	Diminished diarrhea duration Diminished fever duration Diminished vomiting duration	[134]
*Lactobacillus rhamnosus* GG	Diminished risk of nosocomial RV gastroenteritis	[135]
*Lactobacillus. rhamnosus* *Lactobacillus helveticus*	No improvement	[136]
*Lactobacillus rhamnosus* *Lactobacillus helveticus*	No improvement	[137]
*Lactobacillus acidophilus*	No improvement	[138]
*Saccharomyces boulardii*	No improvement	[139]
*Lactobacillus rhamnosus* *Lactobacillus helveticus*	No improvement	[140]
*Lactobacillus rhamnosus* GG	No improvement	[141]
*Lactobacillus rhamnosus* GG	No improvement	[142]
*Lactobacillus paracasei* ST11	No improvement	[143]
*Lactobacillus rhamnosus* GG	No improvement	[144]

^a^ Note that the taxonomy of the genus *Lactobacillus* has suffered recent changes, with the creation of more than 20 new genera [146]. Therefore, some of the strains previously classified as *Lactobacillus* can be ascribed to new genera of lactic acid bacteria, although they are generally recognized as “lactobacilli”.
